# Impact of fracture‐prone implantable cardioverter defibrillator leads on long‐term patient mortality

**DOI:** 10.1002/joa3.12843

**Published:** 2023-03-26

**Authors:** Toshiharu Koike, Morio Shoda, Koichiro Ejima, Daigo Yagishita, Atsushi Suzuki, Shun Hasegawa, Shohei Kataoka, Kyoichiro Yazaki, Satoshi Higuchi, Miwa Kanai, Junichi Yamaguchi

**Affiliations:** ^1^ Department of Cardiology Tokyo Women's Medical University 8‐1 Kawada‐cho, Shinjuku‐ku Tokyo 162‐8666 Japan; ^2^ Clinical Research Division for Heart Rhythm Management, Department of Cardiology Tokyo Women's Medical University 162‐8666 Tokyo 8‐1 Kawada‐cho, Shinjuku‐ku Japan

**Keywords:** advisory lead, implantable cardioverter‐defibrillator lead, lead failure, Linox, mortality

## Abstract

**Background:**

The long‐term relationship between fracture‐prone implantable cardioverter‐defibrillator (ICD) leads and poor prognosis remains unclear in Japanese patients.

**Methods:**

We conducted a retrospective review of the records of 445 patients who underwent implantation of advisory/Linox leads (Sprint Fidelis, 118; Riata, nine; Isoline, 10; Linox S/SD, 45) and non‐advisory leads (Endotak Reliance, 33; Durata, 199; Sprint non‐Fidelis, 31) between January 2005 and June 2012 at our hospital. The primary outcomes were all‐cause mortality and ICD lead failure. The secondary outcomes were cardiovascular mortality, heart failure (HF) hospitalization, and the composite outcome of cardiovascular mortality and HF hospitalization.

**Results:**

During the follow‐up period (median, 8.6 [4.1–12.0] years), there were 152 deaths: 61 (34%) in patients with advisory/Linox leads and 91 (35%) in those with non‐advisory leads. There were 32 ICD lead failures: 27 (15%) in patients with advisory/Linox leads and five (2%) in those with non‐advisory leads. Multivariate analysis for ICD lead failure demonstrated that the advisory/Linox leads had a 6.65‐fold significantly greater risk of ICD lead failure than non‐advisory leads. Congenital heart disease (hazard ratio 2.51; 95% confidence interval 1.08–5.83; *p* = .03) could also independently predict ICD lead failure. Multivariate analysis for all‐cause mortality demonstrated no significant association between advisory/Linox leads and all‐cause mortality.

**Conclusions:**

Patients who have implanted fracture‐prone ICD leads should be carefully followed up for ICD lead failure. However, these patients have a long‐term survival rate comparable with that of patients with non‐advisory ICD leads in Japanese patients.

## Graphical abstract

Patients who were implanted with fracture‐prone ICD leads should be carefully followed up for ICD lead failure. However, such patients have comparable long‐term survival rates with those with non‐advisory ICD leads.

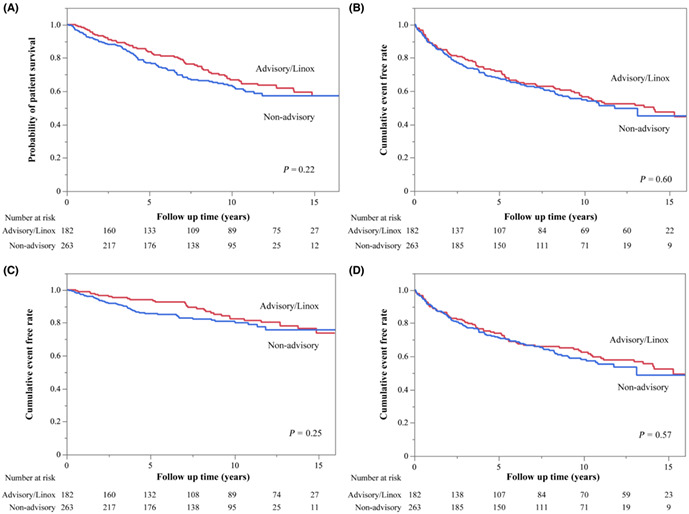

## 1 INTRODUCTION

The role of implantable cardioverter‐defibrillators (ICDs) in reducing mortality from life‐threatening arrhythmias in high‐risk patients has been established.^1^, ^2^ The failure of high‐voltage leads can compromise ICD function, causing inappropriate ICD shocks, defibrillation failure, fatal proarrhythmias, and even death.^3^, ^4^ The Sprint Fidelis (Medtronic, Minneapolis, MN, USA), Riata and Riata ST (St. Jude Medical, Sylmar, CA, USA), and Isoline leads (SORIN CRM SAS, Clamart, France) were withdrawn from the market as a result of an increased rate of lead fracture compared with leads from other manufacturers.^5^, ^6^, ^7^ Linox S/SD leads (Biotronik, Berlin, Germany) also reportedly had unacceptably high rates of lead failure.^8^, ^9^, ^10^ Several previous studies have been published on the relationship between these fracture‐prone ICD leads and mortality compared with non‐advisory leads.^11^, ^12^ However, the follow‐up periods were relatively short (3–4 years).^11^, ^12^ Furthermore, these previous studies were almost entirely from Europe or North America. Data are scarce on the relationship between fracture‐prone ICD leads and mortality in the Asian population compared with non‐advisory leads. Therefore, this study aimed to investigate the long‐term relationship between fracture‐prone ICD leads and poor prognosis (all‐cause mortality and cardiovascular adverse events) in Japanese patients.

## 2 METHODS

We conducted a retrospective review of the records of patients with Sprint Fidelis leads (Medtronic, Minneapolis, MN, USA, models 6948 and 6949), Riata leads (St. Jude Medical, Sylmar, CA, USA, models Riata 1570, Riata 1580, and Riata 1581), Isoline leads (SORIN CRM SAS, Clamart, France, model 2CR‐6), Linox leads (Biotronik, Berlin, Germany, models Linox S, Linox SD, and Linox smart S), Durata leads (St. Jude Medical, Sylmar, CA, USA, models 7120, 7120Q, 7121, 7121Q, 7122, 7122Q, 7130, and 7131), Sprint non‐Fidelis leads (Medtronic, Minneapolis, MN, USA, models 6932, 6942, 6943, 6945 and 6947), and Endotak Reliance G/SG leads (Boston Scientific, models 0148, 0171, 0180, 0181, 0184, 0185, 0296, 0292, and 0293) implanted between January 2005 and June 2012 at Tokyo Women's Medical University Hospital. The device and clinical records of all patients were obtained from our hospital records and the device's remote monitoring system and included information on patient characteristics, clinical history, and various parameters of the ICD leads. The study was approved by the institutional review board of Tokyo Women's Medical University Hospital (ID: 2021‐0192). Because of the retrospective nature of the study, informed consent was not necessary, and the opt‐out method was used through the hospital website.

All study ICD leads were implanted by experienced cardiologists specializing in electrophysiology. ICD leads were implanted via a left‐ or right‐sided cephalic vein by cut‐down, axillary, or subclavian veins using standard puncture techniques. ICD leads were positioned in the right ventricular apex or the ventricular septum. After lead implantation, the R‐wave amplitude, lead impedance, and pacing threshold were evaluated in all patients. After testing, they were connected to the ICD pulse generator, and the incision was closed. After ICD implantation, patients were followed up every 6 months using a remote monitoring system (RMS) or through ambulatory follow‐ups at our hospital. The lead integrity alert (LIA) algorithm was used to detect early lead fractures, as described in a previous report.^13^

In accordance with the previously reported ICD lead survival rates,^5^, ^6^, ^7^, ^8^, ^9^, ^10^, ^14^, ^15^, ^16^ patients were categorized into two groups: patients implanted with fracture‐prone ICD leads (Sprint Fidelis, Riata, Isoline, or Linox leads) and those implanted with non‐advisory leads (Durata, Endotak Reliance, or Sprint non‐Fidelis leads). Linox leads have been reported to have comparable lead survival rates to those of advisory leads.^8^, ^9^, ^10^ Therefore, patients implanted with Linox leads were grouped with those with advisory leads.

ICD lead failure was defined as a lead removed from service because of its inability to meet its performance specifications or otherwise not performing as intended. In line with previously established definitions,^11^, ^14^, ^15^ a lead had failed if it met one or more of the following criteria: (i) a sudden rise in long‐term pacing or high‐voltage impedance, (ii) electrical noise artifacts manifested by non‐physiological signals on the electrogram or by device diagnostics (e.g., non‐physiological short intervals and/or recurrent non‐sustained ventricular tachycardia with intervals <220 ms), and (iii) a failure to sense R‐waves or ineffective electrical therapy caused by an apparent structural lead defect. Cases with pseudofractures, because of the header or connector problems, were excluded at the time of replacement. Functional abnormalities, including exit block and physiological oversensing with an electrically intact lead, were excluded from the definition of ICD lead failure. Lead dislodgement was not considered an ICD lead failure. A lead‐related death was defined as a sudden or unexpected death accompanied by evidence of an ICD lead failure. A group of 100%‐paced patients were categorized by complete heart block in the absence of a stable escape rhythm or 100% pacing in patients without cardiac resynchronization therapy (CRT). The primary outcomes of the study were all‐cause mortality and ICD lead failure. The secondary outcomes were cardiovascular mortality, heart failure (HF) hospitalization, and the composite outcome of cardiovascular mortality and HF hospitalization.

According to the distribution and dispersion, continuous variables were expressed as means ± standard deviations or as medians with interquartile ranges (IQR). The one‐way ANOVA and Kruskal–Wallis tests were used to compare continuous variables between groups. Categorical variables were presented as numbers and percentages and were compared using Pearson's Chi‐squared test and Fisher's exact test, as appropriate. For post‐hoc comparisons between groups, the Bonferroni correction and Steel–Dwass test were used to compare continuous and categorical variables. The incidence of the clinical outcome was assessed using the Kaplan–Meier method, and the significance of differences among groups was compared using the log‐rank test with a Bonferroni correction. The time to lead failure was calculated from the date of ICD lead implantation to ICD lead failure. ICD leads were censored if ICD lead extraction, ICD lead revision, or heart transplantation were conducted, or if the study exit had occurred. Patients were censored if they died or left the study. A Cox proportional hazards model was used to evaluate predictors of the primary outcome via univariate and multivariate analyses. Multivariate analysis was performed according to the results of the univariate analysis. All analyses were performed using JMP 16 (SAS Institute, Cary, NC, USA), and a two‐sided *p* < .05 was considered significant.

## 3 RESULTS

A total of 445 patients (Sprint Fidelis, *n* = 118; Riata, *n* = 9; Isoline *n* = 10; Linox, *n* = 45; Durata, *n* = 199; Endotak Reliance *n* = 33; Sprint non‐Fidelis *n* = 31) who underwent implantation of an ICD lead were included in the study. The baseline characteristics of patients with advisory/Linox leads and non‐advisory leads are shown in Table 1. The median follow‐up period, until the last follow‐up date, in patients with advisory/Linox leads was significantly longer than that in patients with non‐advisory leads. The prevalence of HF and screw‐in lead use in patients with non‐advisory leads were significantly higher than that in patients with advisory/Linox leads. The prevalence of a lead length ≥65 cm in patients with advisory/Linox leads was significantly higher than that in patients with non‐advisory leads. There were significant differences in age and left ventricular ejection fraction (LVEF) between the two groups. Table S1 demonstrates baseline patient characteristics categorized by ICD lead types.

**TABLE 1:** Baseline patient characteristics.

	Advisory/Linox (*n* = 182)	Non‐advisory (*n* = 263)	*p*‐value
Median follow‐up period until patient's last follow‐up date (years)	9.7 [4.7–14.1]	7.7 [3.8–11.1]	<.0001
Median follow‐up period until ICD lead removal or revision date (years)	7.1 [3.7–12.8]	7.4 [3.6–10.8]	.22
Age (years)	60 [46–68]	63 [50–71]	.03
Male	132 (73)	193 (73)	.84
BMI (kg/m^2^)	23 [20–25]	22 [20–24]	.07
Indication for ICD			
Primary	126 (69)	176 (67)	.61
Secondary	56 (31)	87 (33)	.61
Heart failure	108 (59)	182 (69)	.03
Atrial lead	162 (89)	238 (94)	.61
CRT device	63 (35)	111 (42)	.11
Underlying cardiac disease			
Ischemic cardiomyopathy	45 (25)	58 (22)	.51
Dilated cardiomyopathy	58 (32)	70 (27)	.23
Hypertrophic cardiomyopathy	28 (15)	41 (16)	.95
ARVC	5 (3)	8 (3)	.86
Congenital heart disease	15 (8)	14 (5)	.22
Valve surgery	13 (7)	28 (11)	.21
History of stroke	7 (4)	7 (3)	.48
LVEF (%)	38 [26–54]	32 [22–48]	.003
Atrial fibrillation	64 (35)	72 (27)	.08
Creatinine	0.93 [0.78–1.15]	0.93 [0.74–1.35]	.69
COPD	2 (1)	4 (2)	.70
100% paced except for CRT	29 (16)	29 (11)	.13
ICD lead implantation details			.81
New implant	174 (96)	254 (96)	
Additional implant	6 (3)	6 (2)	
Replacement	2 (1)	3 (1)	
Access vein for ICD lead			.001
Cephalic	101 (55)	173 (66)	
Axillary	12 (7)	2 (1)	
Subclavian	69 (38)	88 (33)	
Left‐sided device implant	171 (94)	249 (95)	.75
Lead length ≥ 65 cm	151 (83)	178 (68)	.0003
Screw‐in lead	164 (90)	253 (96)	.01
Total number of leads at implantation	2 [2–3]	2 [2–3]	.69
Previous lead implantation	34 (19)	39 (15)	.28

*Note*: Data are given as *n* (%), or as median [interquartile range].

Abbreviations: ARVC, arrhythmogenic right ventricular cardiomyopathy; BMI, body mass index; COPD, chronic obstructive pulmonary disease; CRT, cardiac resynchronization therapy; ICD, implantable cardioverter‐defibrillator; LVEF, left ventricular ejection fraction.

A total of 152 patients (34%) died during the median follow‐up of 8.6 years [IQR; 4.1–12.0 years] from the following causes: cardiac issues [*n* = 76], infections [*n* = 12], malignancies [*n* = 8], gastrointestinal deaths [*n* = 8], renal failures [*n* = 7], strokes [*n* = 2], acute respiratory failure [*n* = 1], acute limb ischemia [*n* = 1], trauma [*n* = 1], and other causes [*n* = 36]. The outcomes of cardiovascular mortality, HF hospitalization, and the composite outcome of cardiovascular mortality and HF hospitalization occurred in 76, 164, and 187 patients, respectively. During the follow‐up, until ICD lead removal or revision date, ICD lead failure, ICD lead extraction, ICD appropriate shocks, and ICD inappropriate shocks occurred in 32, 37, 112, and 67 patients, respectively. Sixty‐nine patients experienced ICD inappropriate shocks until the last follow‐up examination (67 patients until ICD lead removal or revision date). The reasons for ICD inappropriate shock were: ICD lead failure (*n* = 13), atrial tachyarrhythmia (*n* = 43), sinus tachycardia (*n* = 9), and other reasons (*n* = 10). This does not match the total number of patients who experienced ICD inappropriate shocks because some patients experienced ICD inappropriate shocks for multiple reasons. RMS induction could be assessed in 382 patients (86%) and was unknown in the other 63 patients (14%). Among the 382 patients, the RMS induction rate before experiencing ICD inappropriate shocks was 75% (*n* = 287). During the follow‐up period, the rate of ICD inappropriate shock was significantly lower in patients on RMS (*n* = 28 [10%]) than that in those without RMS (*n* = 38 [40%]; *p* < .0001).

The fracture sign and lead‐related deaths in patients with ICD lead failure for each ICD lead type are shown in Table 2. During follow‐up, until ICD lead removal or revision date, we identified ICD lead failure in 20 Sprint Fidelis leads (17%), 1 Riata lead (11%), 1 Isoline lead (10%), 5 Linox leads (11%), 4 Durata leads (3%), and 1 Sprint non‐Fidelis lead (3%). The median interval from implantation to ICD lead failure was 6.0, 12.4, 9.3, 8.3, 4.7, and 9.5 years with Sprint Fidelis, Riata, Isoline, Linox, Durata, and Sprint non‐Fidelis leads, respectively. In 22 leads, a sudden rise in long‐term pacing or high‐voltage impedance was found. In 16 leads, electrical noise artifacts were found, and 12 patients experienced inappropriate shocks because of the electrical noise artifacts. The LIA was activated in 20 patients and prevented inappropriate shocks in 13 of them. No lead‐related death occurred in any patient with ICD lead failure. An externalized conductor was found in a fractured Riata lead.

**TABLE 2:** Fracture sign and ICD lead‐related death in patients with ICD lead failure.

	Sprint fidelis (*n* = 20)	Riata (*n* = 1)	Isoline (*n* = 1)	Linox (*n* = 5)	Durata (*n* = 4)	Sprint non‐fidelis (*n* = 1)
Time to ICD lead failure (years)	6.0 [4.7–8.7]	12.4	9.3	8.3 [2.5–10.6]	4.7 [1.4–7.6]	9.5
A sudden rise in long‐term pacing or high‐voltage impedance	13 (65)	1 (100)	1 (100)	2 (40)	4 (100)	1 (100)
Electrical noise artifacts	13 (65)	0 (0)	0 (0)	3 (60)	0 (0)	1 (100)
Inappropriate shocks because of electrical noise artifacts	9 (45)	0 (0)	0 (0)	3 (60)	0 (0)	1 (100)
Failure to sense R‐wave or ineffective electrical therapy because of an apparent structural lead defect	1 (5)	0 (0)	0 (0)	0 (0)	0 (0)	1 (100)
Lead integrity alert	17 (85)	0 (0)	1 (100)	1 (20)	1 (25)	0 (0)
ICD lead‐related death	0 (0)	0 (0)	0 (0)	0 (0)	0 (0)	0 (0)

*Note*: Data are given as *n* (%), or as the median [interquartile range].

Abbreviation: ICD, implantable cardioverter‐defibrillator.

Table 3 shows the hazard ratio (HR) of ICD lead failure, until the ICD lead removal or revision date, based on the univariate analysis. Advisory/Linox, Sprint Fidelis, and Linox leads were significantly associated with increased ICD lead failure. Other devices and patient factors associated with increased ICD lead failure in univariate analysis were congenital heart disease and the total number of leads at implantation. Multivariate analysis was performed according to the results of the univariate analysis. Advisory/Linox leads had a 6.65‐fold higher risk of ICD lead failure than non‐advisory leads (HR, 6.65; 95% confidence interval [CI], 2.55–17.4; *p* = .0001). Moreover, congenital heart disease could also independently predict ICD lead failure (HR, 2.51; 95% CI, 1.08–5.83; *p* = .03).

**TABLE 3:** Univariate and multivariate predictors of ICD lead failure.

Variables	Univariate		Multivariate	
	HR and 95% CI	*p*‐value	HR and 95% CI	*p*‐value
Advisory/Linox vs. non‐advisory	7.27 [2.79–18.9]	<.0001	6.65 [2.55–17.4]	.0001
ICD lead type		.001		
Sprint Fidelis vs. non‐advisory	8.70 [3.25–23.3]	<.0001		
Riata vs. non‐advisory	6.34 [0.74–54.6]	.09		
Isoline vs. non‐advisory	5.29 [0.62–45.3]	.13		
Linox vs. non‐advisory	4.71 [1.37–16.3]	.01		
Age (years)	0.99 [0.97–1.01]	.34		
Male	0.80 [0.39–1.67]	.56		
BMI (kg/m^2^)	1.01 [0.92–1.10]	.79		
ICD indication for primary prevention	0.66 [0.33–1.34]	.25		
CRT	0.53 [0.22–1.30]	.17		
Ischemic cardiomyopathy	1.68 [0.75–3.76]	.20		
Dilated cardiomyopathy	0.63 [0.26–1.53]	.31		
Hypertrophic cardiomyopathy	0.29 [0.07–1.20]	.09		
ARVC	0.91 [0.12–6.64]	.91		
Congenital heart disease	3.16 [1.37–7.32]	.007	2.51 [1.08–5.83]	.03
100% paced except for CRT	0.70 [0.21–2.30]	.56		
ICD lead inserted at first ICD implantation	0.56 [0.14–2.37]	.44		
ICD lead access via subclavian vein	1.48 [0.73–2.99]	.28		
Left‐sided device implant	0.53 [0.16–1.73]	.29		
ICD lead length ≥ 65 cm	1.18 [0.51–2.73]	.70		
Previous lead implantation	0.84 [0.29–2.39]	.74		
Total number of leads at implantation	0.56 [0.32–0.95]	.04	0.59 [0.32–1.02]	.07

Abbreviations: ARVC, arrhythmogenic right ventricular cardiomyopathy; BMI, body mass index; CI, confidence interval; CRT, cardiac resynchronization therapy; HR, hazard ratio; ICD, implantable cardioverter‐defibrillator.

**FIGURE 1:**
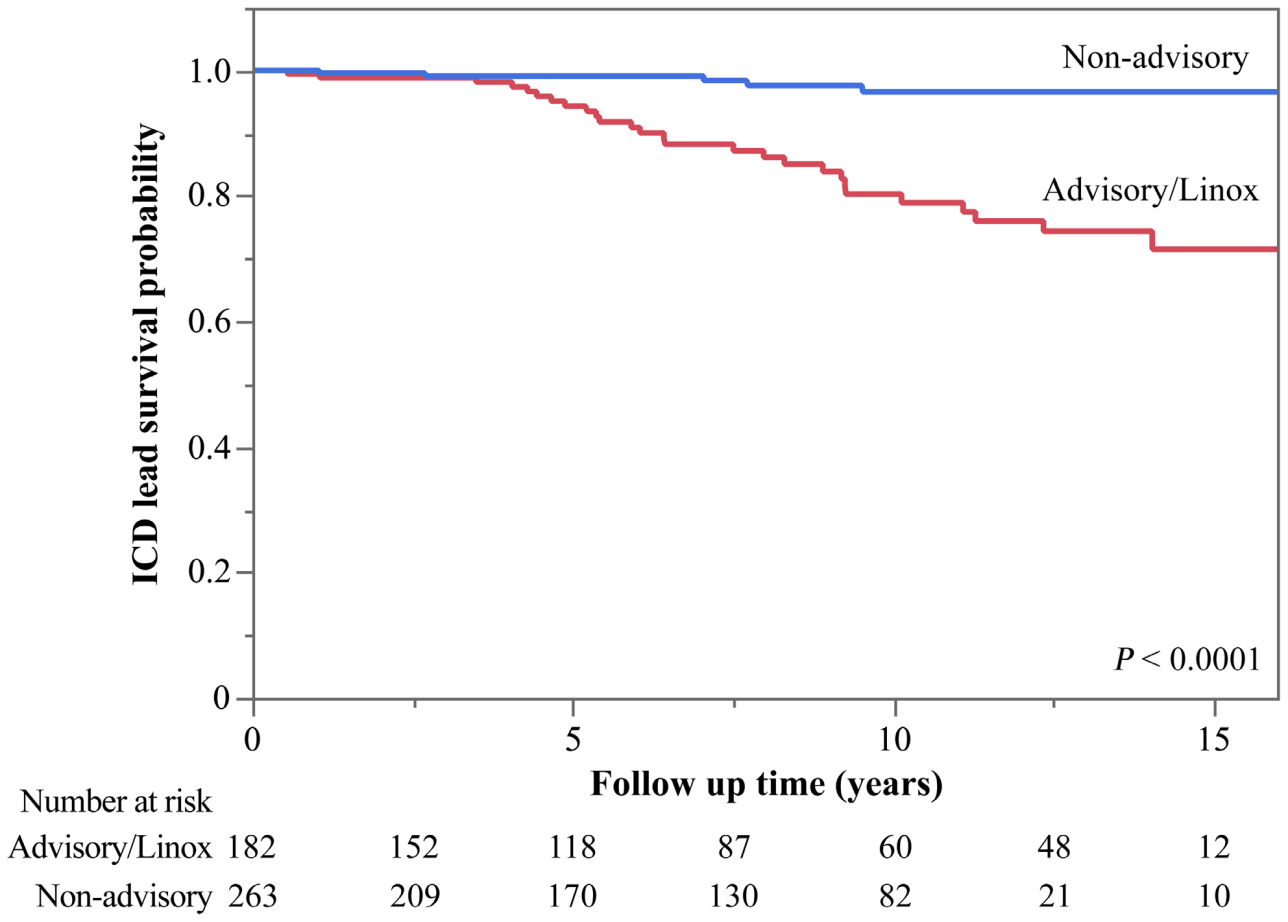
Kaplan–Meier curve of cumulative ICD lead survival rates for advisory/Linox leads and non‐advisory leads. ICD, implantable cardioverter‐defibrillator.

Figure 1 shows the Kaplan–Meier curves of the ICD lead survival rates for advisory/Linox leads and non‐advisory leads. During the follow‐up period, until the ICD lead removal or revision date, ICD lead failure occurred more frequently in patients with advisory/Linox leads than in patients with non‐advisory leads (*p* < .0001). The 10‐year ICD lead survival rates were 80% and 97% for advisory/Linox leads and non‐advisory leads, respectively. Figure S1 shows the Kaplan–Meier curves of ICD lead outcome‐free survival rates for each ICD lead type. There were significant differences in ICD lead survival rates among the different ICD lead types (*p* = .0002). In particular, Sprint Fidelis leads fractured more frequently than all other non‐advisory leads.

Table 4 shows the HR of all‐cause mortality based on the univariate and multivariate analyses. In the univariate analyses, advisory/Linox leads and an ICD lead type were not significantly associated with all‐cause mortality. Conversely, age, CRT, HF, ischemic cardiomyopathy, congenital heart disease, LVEF, atrial fibrillation (AF), elevated creatinine, ICD appropriate shock, and ICD inappropriate shock were associated with increased all‐cause mortality in the univariate analyses. Based on the results of univariate analyses, multivariate analyses were performed in two models with variables that were considered to be related to all‐cause mortality. Model 1 considered whether fracture‐prone ICD leads were implanted, while Model 2 considered the influence of fracture‐prone ICD lead type (Sprint Fidelis, Riata, Isoline, or Linox) on all‐cause mortality. The advisory/Linox leads and all fracture‐prone ICD lead types were not significantly associated with increased all‐cause mortality compared with non‐advisory leads. However, advanced age, elevated creatinine levels, HF, and AF were independent predictors of all‐cause mortality. In the multivariate analyses for all‐cause mortality, including each reason for ICD inappropriate shock, the reasons for ICD inappropriate shock were not significantly associated with all‐cause mortality (Table S2).

**TABLE 4:** Univariate and multivariate predictors of mortality until the last follow‐up date.

Variables	Univariate		Multivariate			
			Model 1		Model 2	
	HR and 95% CI	*p*‐value	HR and 95% CI	*p*‐value	HR and 95% CI	*p*‐value
Advisory/Linox vs. non‐advisory	1.23 [0.89–1.71]	.22	0.93 [0.66–1.31]	.66		
ICD lead type		.14				.34
Sprint Fidelis vs. non‐advisory	1.00 [0.70–1.43]	1.00			1.14 [0.78–1.68]	.50
Riata vs. non‐advisory	0.57 [0.14–2.31]	.43			0.68 [0.17–2.81]	.60
Isoline vs. non‐advisory	0.95 [0.35–2.59]	.92			0.69 [0.25–1.91]	.68
Linox vs. non‐advisory	0.40 [0.19–0.82]	.01			0.53 [0.25–1.11]	.09
Age (years)	1.04 [1.02–1.05]	<.0001	1.03 [1.01–1.04]	.0003	1.03 [1.01–1.04]	.001
Male	1.32 [0.91–1.93]	.15				
BMI (kg/m^2^)	0.97 [0.93–1.01]	.17				
ICD indication for primary prevention	1.19 [0.84–1.69]	.34				
Atrial lead	1.36 [0.75–2.45]	.31				
CRT	2.17 [1.58–2.98]	<.0001	1.05 [0.72–1.54]	.80	1.16 [0.78–1.73]	.45
Heart failure	3.90 [2.54–6.00]	<.0001	2.38 [1.38–4.11]	.002	2.33 [1.36–4.00]	.002
Ischemic cardiomyopathy	1.99 [1.41–2.81]	<.0001	1.29 [0.89–1.87]	1.29	1.29 [0.89–1.87]	.17
Dilated cardiomyopathy	1.21 [0.86–1.70]	.28				
Hypertrophic cardiomyopathy	0.73 [0.46–1.18]	.20				
ARVC	1.30 [0.58–2.95]	.52				
Congenital heart disease	0.38 [0.15–0.92]	.03	0.97 [0.37–2.49]	.94	0.96 [0.37–2.49]	.93
Valve surgery	1.43 [0.86–2.37]	.17				
History of stroke	1.70 [0.80–3.63]	.17				
LVEF (%)	0.97 [0.96–0.98]	<.0001	0.99 [0.98–1.01]	.27	0.99 [0.98–1.01]	.30
Atrial fibrillation	2.10 [1.52–2.90]	<.0001	1.79 [1.27–2.53]	.001	1.75 [1.24–2.48]	.002
Creatinine	1.15 [1.08–1.21]	<.0001	1.16 [1.07–1.23]	<.0001	1.15 [1.07–1.23]	<.0001
COPD	1.38 [0.44–4.34]	.58				
100% paced except for CRT	1.09 [0.69–1.73]	.71				
ICD lead inserted at first ICD implant	1.93 [0.62–6.05]	.26				
ICD lead access via subclavian vein	1.13 [0.81–1.58]	.46				
Left‐sided device implant	0.97 [0.50–1.91]	.93				
ICD lead length ≥ 65 cm	0.95 [0.66–1.37]	.80				
Screw‐in lead	2.24 [0.92–5.47]	.08				
Previous lead implantation	1.37 [0.91–2.06]	.13				
CIED infection	1.08 [0.51–2.32]	.83				
ICD appropriate shock	1.59 [1.14–2.22]	.006	1.36 [0.96–1.92]	.08	1.32 [0.93–1.87]	.12
ICD inappropriate shock	0.50 [0.30–0.85]	.01	0.69 [0.41–1.18]	.23	0.67 [0.39–1.15]	.15
ICD lead extraction	0.67 [0.35–1.28]	.23				

Abbreviations: ARVC, arrhythmogenic right ventricular cardiomyopathy; BMI, body mass index; CI, confidence interval; CIED, cardiac implantable electronic device; COPD, chronic obstructive pulmonary disease; CRT, cardiac resynchronization therapy; HR, hazard ratio; LVEF, left ventricular ejection fraction; ICD, implantable cardioverter‐defibrillator.

**FIGURE 2:**
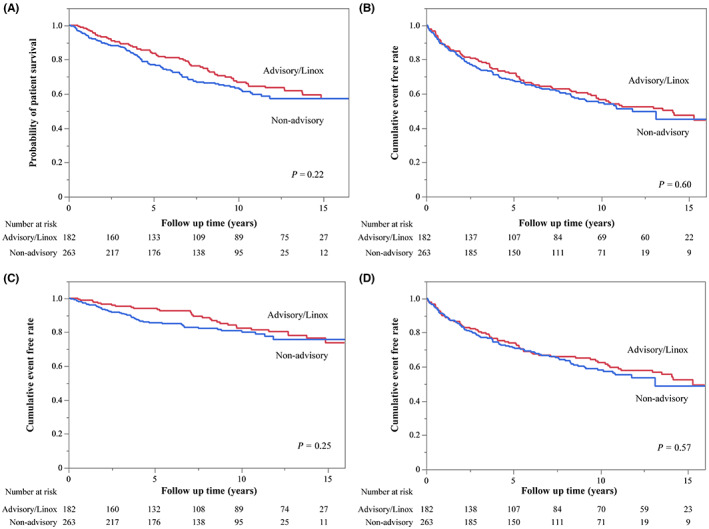
Difference in outcome‐free survival rates between patients with advisory/Linox leads and those with non‐advisory leads. Kaplan–Meier curves showing the differences in the cumulative rates of the following clinical outcomes until the last follow‐up date: (A) All‐cause mortality, (B) Composite outcome, (C) Cardiovascular mortality, (D) Heart failure hospitalization. ICD, implantable cardioverter‐defibrillator.

Figure 2 shows the Kaplan–Meier curve analysis and illustrates the differences in the cumulative rates of clinical outcomes between patients with advisory/Linox leads and those with non‐advisory leads. There were no significant differences between the two groups in the incidence of all‐cause mortality, the outcome of cardiovascular mortality, HF hospitalization, and the composite outcome of cardiovascular mortality and HF hospitalization (Figures 2A–D). Figure S2 shows the Kaplan–Meier curves of all‐cause mortality and the composite outcome of cardiovascular mortality and HF hospitalization categorized by ICD lead type. Among all the ICD lead types, there was no significant difference in all‐cause mortality or the composite outcome of cardiovascular mortality and HF hospitalization.

## 4 DISCUSSION

Our study has four main findings. First, the multivariate analysis for ICD lead failure found that advisory/Linox leads and congenital heart disease were significantly associated with ICD lead failure in the long term. Second, no lead‐related death occurred in any patient with confirmed ICD lead failure. Third, the multivariate analysis for all‐cause mortality found that using fracture‐prone ICD leads did not affect the incidence of all‐cause mortality. Conversely, having an advanced age, elevated creatinine level, HF, and AF independently predicted all‐cause mortality. Finally, in the Kaplan–Meier analysis for the incidence of adverse clinical events other than ICD lead failure, patients implanted with fracture‐prone ICD leads did not have more frequent all‐cause mortality and cardiovascular adverse events (cardiovascular mortality and/or HF hospitalization) than those with non‐advisory leads.

ICDs perform a critical lifesaving function to prevent sudden cardiac death.^1^, ^2^ Conversely, ICD lead failure can result in significant clinical adverse events, including ICD inappropriate shock therapy and even death.^3^, ^4^, ^17^ In particular, patients who were implanted with advisory leads could be exposed to a more frequent risk of ICD lead failure than those who received non‐advisory leads.^5^, ^6^, ^7^, ^14^, ^15^, ^16^, ^18^, ^19^, ^20^ With respect to Linox leads, not advisory leads, several previous studies have suggested that the unacceptably high rate of lead failure contradicts the self‐reported data from the manufacturer.^8^, ^9^, ^10^ Nevertheless, few studies have evaluated whether using fracture‐prone ICD leads to increased mortality compared with using non‐advisory leads.^11^, ^12^

Some previous studies have shown that Sprint Fidelis leads did not increase all‐cause mortality in the relatively short period of 2.9–3.2 years, compared with Sprint Quatro leads.^11^, ^12^ Furthermore, these studies were limited to one type of advisory lead.^11^, ^12^ It should be noted that Hauser et al. demonstrated that abnormal high‐voltage impedances were the hallmark of Riata lead failure, often resulting in failure to defibrillate and lead‐related death.^21^ In contrast, Iftikhar A et al. demonstrated that no lead‐related death had been identified in patients implanted with Riata leads for mean follow‐up periods of approximately 4.5 years.^22^ Thus, there are conflicting reports regarding Riata lead‐related deaths.^21^, ^22^ Regarding Isoline and Linox leads, to the best of our knowledge, no study has evaluated the relationship between these fracture‐prone ICD leads and all‐cause mortality compared with non‐advisory leads in detail.

The present study included various fracture‐prone ICD leads, comprising those by all manufacturers that have produced fracture‐prone ICD leads to date. In the univariate analysis (Table 4), advisory/Linox leads and ICD lead types were not significantly associated with all‐cause mortality. The multivariate analysis demonstrated that advisory/Linox ICD leads did not independently increase all‐cause mortality in the long term (median follow‐up period: 9.7 and 7.7 years in patients with advisory/Linox leads and in those with non‐advisory leads, respectively; Table 4). In addition, no patient with advisory/Linox leads died because of ICD lead failure in the long term. Patients implanted with fracture‐prone ICD leads had adverse cardiovascular events (cardiovascular mortality and/or HF hospitalization) comparable with those of patients implanted with non‐advisory leads (Figures 2B–D). The median follow‐up periods in patients implanted with Sprint Fidelis, Riata, Isoline, and Linox leads were 8.5, 6.0, 11.5, and 11.5 years, respectively Table S1. These follow‐up periods were longer than those in the previous studies that investigated the relationship between fracture‐prone ICD leads and mortality.^11^, ^12^ Furthermore, patients continued follow‐up after ICD lead removal or revision. Therefore, the design of the current study allowed the consideration of the impact of ICD inappropriate shocks and ICD lead extraction, which were often accompanied by ICD lead failure, on prognosis.

In the multivariate analysis, having an advanced age, a decline in renal function, HF, and AF were independent risk factors for all‐cause mortality (Table 4). Several previous studies have shown that patients implanted with ICD who have advanced age, or AF or HF, have a poorer prognosis than those without these risk factors.^23^, ^24^ Hager et al. showed that chronic kidney disease was an independent predictor of mortality in patients who underwent ICD implantation for primary prevention.^25^ These findings support our results.

ICD inappropriate shock is associated with increased mortality.^17^ In the present study, multivariate analyses for all‐cause mortality demonstrated that the reasons for ICD inappropriate shock were not significantly associated with all‐cause mortality Table S2. Kleeman et al. also demonstrated that ICD inappropriate shocks accompanied by ICD lead failure were not significantly associated with poor prognosis.^26^ Even if ICD inappropriate shocks were accompanied by ICD lead failure, the inappropriate ICD shock therapy might not affect long‐term mortality with appropriate ICD lead revision. Further research on the impact of ICD inappropriate shocks accompanied by ICD lead failure is needed.

In the present study, although RMS induction status in some patients was unknown, the rate of ICD inappropriate shock was significantly lower in patients using the RMS than that in those who did not use the RMS during the follow‐up period. Similarly, the ECOST trial (Laurence et al.) demonstrated that RMS for ICDs lowered the number of ICD inappropriate shocks.^27^ Furthermore, after a follow‐up period of approximately 2 years, the same study showed that the proportion of patients experiencing ICD inappropriate shocks was 52% lower in patients that used the RMS than in those who did not.^27^

Several previous studies have demonstrated that the high rate of Sprint Fidelis failure and the risk of lead failure increase with time.^18^, ^19^, ^20^ Moreover, some earlier studies have shown that Riata, Isoline, and Linox leads had unacceptably higher rates of lead failure than other leads.^5^, ^6^, ^7^, ^16^, ^18^ Similarly, the present study demonstrated that advisory/Linox leads had a 6.65‐fold greater risk of ICD lead failure than non‐advisory leads, after adjusting for cofounders that were considered to be related to ICD lead failure (Table 3). Moreover, Sprint Fidelis and Linox leads had a significantly greater risk of ICD lead failure than that non‐advisory leads (Table 3). In contrast, Riata and Isoline leads did not have a significantly greater risk of ICD lead failure in the present study; however, this may have been because of the smaller number of patients implanted with Riata leads (*n* = 9) and Isoline leads (*n* = 10). The small patient number may have resulted in Riata and Isoline leads not demonstrating a significantly greater risk of ICD lead failure than non‐advisory leads.

In Durata lead failures, the frequencies of electrical noise artifacts and ICD inappropriate shocks were lower than those when using the Sprint Fidelis or Linox leads (Table 2). Although no direct comparison between the Durata leads and the other two leads was made, single‐coil Durata models are less likely to develop internal insulation breaches than dual‐coil Durata models.^28^ Internal insulation breaches could disrupt the silicone, abrade the fluorine‐based protective coating, and short‐circuit conductors to each other or to the shock coils.^28^ The internal insulation breaches may present as electrical noise artifacts with ICD inappropriate shocks.^28^ In the present study, the models of fractured Durata leads were the Durata 7120 (*n* = 1), 7122 (*n* = 2), and 7122Q (*n* = 1). Single‐coil Durata models (7122 and 7122Q) accounted for the majority of Durata lead failures. This might have resulted in less ICD inappropriate shocks accompanied by Durata lead failures in the present study. Hauser et al. demonstrated that fracture of the pace‐sense conductor was the predominant failure mechanism in Sprint Fidelis lead failures. ^29^ Lead fracture of the pace‐sense conductor may instantaneously lead to the incidence of electrical noise artifacts and ICD inappropriate shocks. This lead failure mechanism may explain the different clinical manifestation in Sprint Fidelis lead failures. To our knowledge, no study has analyzed the mechanisms of Linox lead failures. Further research for the mechanism of Linox lead failures is needed.

Furthermore, in the present study, congenital heart disease was also an independent predictor of ICD lead failure. ICD lead failures occur at higher rates in patients who are young and have congenital heart diseases.^30^, ^31^ In the present study, the median age of patients with congenital heart disease was 37 [IQR, 27–47] years, which was significantly less than that of patients without congenital heart disease which was 62 [IQR, 52–70] years. Although having an advanced age was not significantly associated with ICD lead failure in the present study, the high proportion of young patients with congenital heart disease may have resulted in congenital heart disease being an independent predictor of ICD lead failure.

Our study had some limitations. First, the low incidence of ICD lead failure (*n* = 32) might have resulted in a statistically underpowered analysis of lead failure. Hence, in the multivariate analysis, we could not adequately eliminate the influence of unmeasured cofounders while determining the independent predictors of ICD lead failure. Second, the number of patients implanted with Riata and Isoline leads was relatively small. In the present study, ICD lead failure was identified in only one patient each with Riata or Isoline leads. Therefore, the impact of Riata or Isoline leads on long‐term prognosis in the Japanese population may require further investigation. However, in the present study, 152 deaths occurred during the follow‐up period, which were sufficient to enable an accurate analysis regarding all‐cause mortality. Third, this study did not evaluate all models of fracture‐prone ICD leads. Therefore, further research is needed on the models of fracture‐prone ICD leads that were not included in this study. Fourth, similar to previous studies,^11^, ^12^ device interrogations were not necessarily obtained from all patients. Therefore, we were unable to adjudicate the probability of death because of the ICD lead failure in some patients. Finally, the study was a retrospective analysis from a single center.

## 5 CONCLUSIONS

Using advisory/Linox leads and having congenital heart disease independently predicted ICD lead failure. However, advisory/Linox leads were not associated with increased all‐cause mortality in extended long‐term follow‐up in Japanese patients. Patients who were implanted with fracture‐prone ICD leads should be carefully followed up for ICD lead failure. However, such patients have comparable long‐term survival rates as those with non‐advisory ICD leads.

## AUTHOR CONTRIBUTIONS

Toshiharu Koike: Conceptualization, design, data curation and acquisition, investigation, data interpretation, formal analysis, and writing—original draft preparation. Morio Shoda: Writing—review and editing, data interpretation. Koichiro Ejima: Writing—reviewing and editing, data interpretation. Daigo Yagishita: Writing—reviewing and editing, data interpretation. Atsushi Suzuki: Writing—reviewing and editing, data interpretation. Shun Hasegawa: Data curation. Shohei Kataoka: Data curation. Kyoichiro Yazaki: Data curation. Satoshi Higuchi: Data curation. Miwa Kanai: Data curation. Junichi Yamaguchi: Data curation. All authors approved the final version of the article.

## CONFLICT OF INTEREST STATEMENT

None.

## INFORMED CONSENT

As a result of the retrospective nature of the study, informed consent was not necessary, and the opt‐out method was used through the hospital website.

## Supporting information


Figure S1

Figure S2

Table S1

Table S2
