# Supplementary figures and images for: Impact of fracture‐prone implantable cardioverter defibrillator leads on long‐term patient mortality
Source: J Arrhythm. 2023 Mar 26;39(3):454–63. doi: 10.1002/joa3.12843 (PMC10264742; doi:10.1002/joa3.12843)

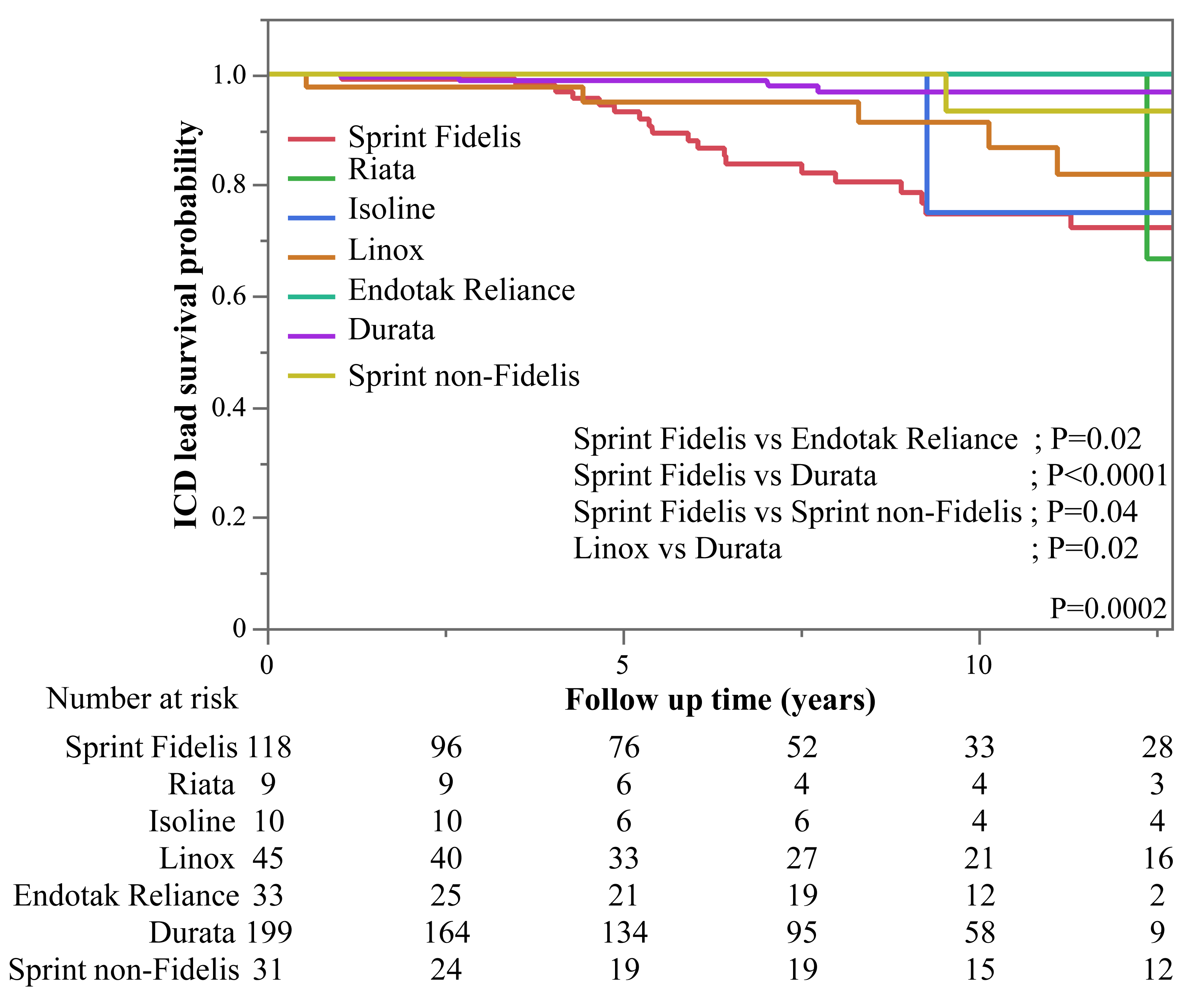

Supplement: Supplementary file 1 — Figure S1 [file JOA3-39-454-s001.tif]

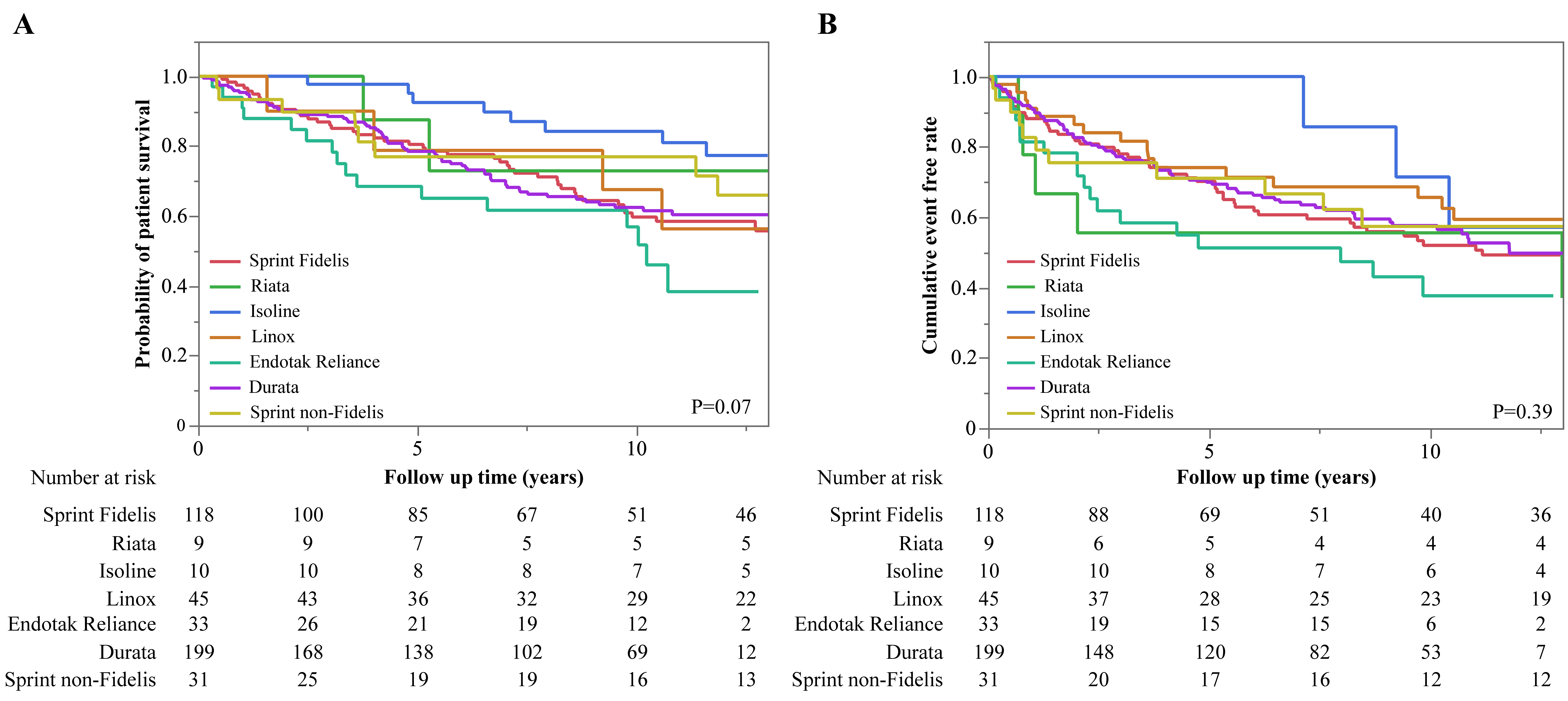

Supplement: Supplementary file 2 — Figure S2 [file JOA3-39-454-s004.tif]
