# Supplementary material for: Impact of fracture‐prone implantable cardioverter defibrillator leads on long‐term patient mortality
Source: J Arrhythm. 2023 Mar 26;39(3):454–63. doi: 10.1002/joa3.12843 (PMC10264742; doi:10.1002/joa3.12843)
Supplement: Supplementary file 3 — Table S1 [file JOA3-39-454-s002.docx]

**Supplementary Table 1 Baseline patient characteristics for each ICD lead type**

|  | **Sprint Fidelis (n=118)** | **Riata (n=9)** | **Isoline (n=10)** | **Linox (n=45)** | **Endotak Reliance (n=33)** | **Durata (n=199)** | **Sprint non-Fidelis (n=31)** | ***P* value** |
| --- | --- | --- | --- | --- | --- | --- | --- | --- |
| Median follow-up period until last follow-up date (years) | 8.5 [4.2-14.8] | 6.0 [3.8-14.5] | 11.5 [3.7-13.6] | 11.5 [6.5-13.6] ◯◯□□□ | 8.7 [2.8-10.3] ## | 7.5 [4.2-10.9] ### | 9.4 [2.8-16.9] | 0.001 |
| Median follow-up period until last ICD lead removal or revision date (years) | 6.7 [3.5-12.0] | 5.2 [3.7-13.8] | 6.6 [2.7-13.6] | 9.1 [4.8-13.2] | 8.7 [2.8-10.3] | 7.1 [4.1-10.6] | 9.4 [2.1-16.9] | 0.38 |
| Age (years) | 62 [47-69] | 60 [42-69] | 61 [52-67] | 56 [39-64] | 62 [55-69] | 63 [48-71] | 62 [50-70] | 0.12 |
| Male | 85 (72) | 7 (69) | 9 (90) | 31 (69) | 22 (67) | 146 (73) | 25 (81) | 0.73 |
| BMI (kg/m^2^) | 22 [20-25] | 22 [20-26] | 24 [20-29] | 23 [20-25] | 21 [20-23] | 22 [20-25] | 22 [20-24] | 0.53 |
| Indication for ICD |  |  |  |  |  |  |  |  |
| Primary | 74 (63) †◯◇◇◇ | 9 (100) * ◇◇◇ | 8 (80) ◇◇◇ | 35 (78) ◇◇◇ | 27 (82) *◇◇◇ | 143 (72) ◇◇◇ | 6 (19) ***†††‡‡‡ ###◯◯◯□□□ | <0.0001 |
| Secondary | 44 (37) †◯◇◇◇ | 0 (0)* ◇◇◇ | 2 (20) ◇◇◇ | 10 (2) ◇◇◇ | 6 (18) *◇◇◇ | 56 (28) ◇◇◇ | 25 (81) ***†††‡‡‡ ###◯◯◯□□□ | <0.0001 |
| Atrial lead | 102 (86) | 9 (100) | 9 (90) | 42 (93) | 30 (91) | 181 (91) | 27 (87) | 0.77 |
| Heart failure | 69 (58) ◯◯◯ | 7 (78) | 7 (70) ◯ | 25 (56) ◯◯◯ | 31 (94) ***‡ ###□□◇◇◇ | 137 (69) ◯◯◇◇ | 14 (45) ◯◯◯□□ | 0.0006 |
| CRT device | 30 (25) ††‡‡‡◯◯◯□□ | 6 (67) **◇◇◇ | 9 (90) ***##□□◇◇◇ | 18 (40) ‡‡◯◯◇◇ | 24 (73) ***##□□◇◇◇ | 84 (42) **‡◯◯◇◇◇ | 3 (10) †††‡‡‡##◯◯◯□□□ | <0.0001 |
| Underlying cardiac disease |  |  |  |  |  |  |  |  |
| Ischemic cardiomyopathy | 30 (25) | 3 (33) | 4 (40) | 8 (18) | 6 (18) | 46 (23) | 6 (19) | 0.69 |
| Dilated cardiomyopathy | 33 (28) | 4 (44) | 3 (30) | 18 (40) | 13 (39) | 52 (26) | 5 (16) | 0.18 |
| Hypertrophic cardiomyopathy | 19 (16) | 1 (11) | 0 (0) | 8 (18) | 5 (15) | 30 (15) | 6 (19) | 0.86 |
| ARVC | 4 (3) | 0 (0) | 0 (0) | 1 (2) | 0 (0) | 5 (3) | 3 (10) | 0.45 |
| Congenital heart disease | 10 (8) | 0 (0) | 1 (10) | 4 (9) | 0 (0) | 13 (7) | 1 (3) | 0.55 |
| Valve surgery | 11 (9) | 1 (11) | 0 (0) | 1 (2) | 4 (12) | 21 (11) | 3 (10) | 0.57 |
| History of stroke | 6 (43) | 0 (0) | 0 (0) | 1 (7) | 2 (14) | 4 (29) | 1 (7) | 0.60 |
| LVEF | 39 [27-56] ◯◯□ | 29 [14-35] | 36 [25-41] | 40 [27-54] ◯ | 24 [19-34]**#◇◇◇ | 33 [23-47] *◇ | 48 [30-60] ◯◯◯□ | <0.0001 |
| Atrial fibrillation | 47 (40) | 1 (11) | 2 (20) | 14 (31) | 9 (27) | 52 (26) | 11 (35) | 0.16 |
| Creatinine | 0.98 [0.77-1.27] | 0.97 [0.85-1.15] | 1.11 [0.79-1.54] | 0.91 [0.70-1.13] | 0.92 [0.72-1.37] | 0.93 [0.74-1.39] | 0.95 [0.76-1.28] | 0.86 |
| COPD | 1 (1) | 0 (0) | 0 (0) | 1 (2) | 0 (0) | 2 (1) | 2 (6) | 0.29 |
| 100% paced except for CRT | 19 (16) | 2 (22) | 2 (20) | 6 (13) | 6 (18) | 20 (10) | 3 (10) | 0.42 |
| ICD lead implantation details |  |  |  |  |  |  |  | 0.93 |
| New implant | 112 (95) | 9 (100) | 10 (100) | 43 (96) | 32 (97) | 191 (96) | 31 (100) |  |
| Additional implant | 4 (1) | 0 (0) | 0 (0) | 2 (4) | 0 (0) | 6 (1) | 0 (0) |  |
| Replacement | 2 (2) | 0 (0) | 0 (0) | 0 (0) | 1 (3) | 2 (1) | 0 (0) |  |
| Access vein for ICD lead |  |  |  |  |  |  |  | 0.008 |
| Cephalic | 73 (62) | 3 (33) | 6 (60) | 19 (42) | 23 (70) | 128 (64) | 22 (71) |  |
| Axillary | 9 (8) | 0 (0) | 0 (0) | 3 (7) | 0 (0) | 2 (1) | 0 (0) |  |
| Subclavian | 36 (31) | 6 (67) | 4 (40) | 23 (51) | 10 (30) | 69 (35) | 9 (29) |  |
| Left-sided device implant | 110 (93) | 7 (78) | 10 (100) | 44 (98) | 31 (94) | 188 (94) | 30 (97) | 0.43 |
| Lead length ≥65 cm | 88 (75) ###◯◯◯ | 9 (100) ◯◯◯ | 9 (90) #◯◯◯ | 45 (100) ***‡◯◯◯□□□◇◇◇ | 1 (3)***††† ‡‡‡###□□□◇◇◇ | 153 (77) ###◯◯◯ | 24 (77) ###◯◯◯ | <0.0001 |
| Screw-in lead | 101 (86) ##□□□ | 8 (89) #□□□ | 10 (100) | 45 (100) ** †◯ ◇◇◇ | 30 (91) #□□□ | 199 (100) *** †††◯◯◯◇◇◇ | 24 (77) ###□□□ | <0.0001 |
| Total number of leads at implantation | 2 [2-3] ◯◯ | 3 [2-4] ◇◇ | 2 [2-3] ◇◇ | 3 [3-3] ◇◇ | 3 [2-3] **◇◇◇□ | 2 [2-3] ◯◇◇ | 2 [2-2] ††‡‡##◯◯◯□□ | <0.0001 |
| Previous lead implantation | 23 (19) | 4 (44) | 1 (10) | 6 (13) | 8 (24) | 30 (15) | 1 (3) | 0.06 |

Data are given as n (%), or as median [interquartile range]. *P<0.05, **P<0.01, ***P<0.001 vs. Sprint Fidelis. †P<0.05, ††P<0.01, †††P<0.001 vs. Riata. ‡P<0.05, ‡‡P<0.01, ‡‡‡P<0.001 vs. Isoline. #P<0.05, ##P<0.01, ###P<0.001 vs. Linox. ◯P<0.05, ◯◯P<0.01, ◯◯◯P<0.001 vs. Endotak reliance. □P<0.05, □□P<0.01, □□□P<0.001 vs. Durata. ◇P<0.05, ◇◇P<0.01, ◇◇◇P<0.001 vs. Sprint non-Fidelis. ARVC, arrhythmogenic right ventricular cardiomyopathy; BMI, body mass index; COPD, chronic obstructive pulmonary disease; CRT, cardiac resynchronization therapy; ICD, implantable cardioverter defibrillator; LVEF, left ventricular ejection fraction.
