# Supplementary material for: Impact of fracture‐prone implantable cardioverter defibrillator leads on long‐term patient mortality
Source: J Arrhythm. 2023 Mar 26;39(3):454–63. doi: 10.1002/joa3.12843 (PMC10264742; doi:10.1002/joa3.12843)
Supplement: Supplementary file 4 — Table S2 [file JOA3-39-454-s003.docx]

**Supplementary Table 2. Univariate and multivariate analyses including the reasons for ICD inappropriate shock on all-cause mortality until the last follow-up date.**

|  | **Model type** | **HR** | **95% CI** | **p-Value** |
| --- | --- | --- | --- | --- |
| No ICD inappropriate shock | Reference | 1.00 |  |  |
| Reasons for ICD inappropriate shock |  |  |  |  |
| ICD lead failure vs. no ICD inappropriate shock | **Univariate** | 0.14 | [0.02-0.99] | 0.05 |
|  | **Multivariate model 1*** | 0.16 | [0.51-1.52] | 0.07 |
| Atrial tachyarrhythmia vs. no ICD inappropriate shock | **Univariate** | 0.69 | [0.39-1.22] | 0.21 |
|  | **Multivariate model 2^#^** | 0.93 | [0.52-1.66] | 0.81 |
| The other reasons vs. no ICD inappropriate shock | **Univariate** | 0.26 | [0.07-1.07] | 0.06 |
|  | **Multivariate model 1*** | 0.53 | [0.13-2.16] | 0.37 |

HR, hazard ratio; CI, confidential interval; ICD, implantable cardioverter defibrillator.

***** Model 1 was adjusted for Advisory/Linox vs. non-advisory, age, cardiac resynchronization therapy (CRT), heart failure (HF), ischemic cardiomyopathy, congenital heart disease, left ventricular ejection fraction (LVEF), atrial fibrillation, creatinine, and ICD appropriate shock.

^#^ Model 2 was adjusted for Advisory/Linox vs. non-advisory, age, CRT, HF, ischemic cardiomyopathy, congenital heart disease, LVEF, creatinine, and ICD inappropriate shock.

In both models, variables that showed significant differences in the univariate analysis for all-cause mortality (Table 4) were included.
